# PGC‐1*α* promotes exercise‐induced autophagy in mouse skeletal muscle

**DOI:** 10.14814/phy2.12698

**Published:** 2016-02-11

**Authors:** Jens F. Halling, Stine Ringholm, Maja M. Nielsen, Peter Overby, Henriette Pilegaard

**Affiliations:** ^1^ Department of BiologyCentre of Inflammation and MetabolismUniversity of CopenhagenCopenhagenDenmark

**Keywords:** Autophagy, exercise, PGC‐1*α*

## Abstract

Recent evidence suggests that exercise stimulates the degradation of cellular components in skeletal muscle through activation of autophagy, but the time course of the autophagy response during recovery from exercise has not been determined. Furthermore, the regulatory mechanisms behind exercise‐induced autophagy remain unclear, although the muscle oxidative phenotype has been linked with basal autophagy levels. Therefore, the aim of this study was to investigate the role of the key regulator of muscle oxidative capacity, PGC‐1*α*, in exercise‐induced autophagy at several time points during recovery. Mice with transgenic muscle‐specific overexpression (TG) or knockout (MKO) of PGC‐1*α* and their respective littermate controls were subjected to a single 1 h bout of treadmill running and euthanized immediately (0 h), 2, 6, and 10 h after exercise. In the PGC‐1*α* MKO strain, quadriceps protein content of the autophagy marker LC3II was increased from 2 h into recovery in lox/lox control, but not in MKO mice. In the PGC‐1*α* TG strain, quadriceps protein content of LC3II was increased from 2 h after exercise in TG, but not in WT. Although AMPK and ACC phosphorylation was increased immediately following exercise, the observed exercise‐induced autophagy response was not associated with phosphorylation of the AMPK‐target ULK1. However, lower protein carbonyl content was observed in lox/lox and TG mice after exercise coinciding with the increased LC3 lipidation. In conclusion, the present results suggest a role of skeletal muscle PGC‐1*α* in coordinating several exercise‐induced adaptive responses including autophagic removal of damaged cellular components.

## Introduction

Physical activity stimulates several metabolic adaptive responses leading to increased oxidative function in skeletal muscle. Studies on exercise‐induced adaptive responses in skeletal muscle have largely focused on the biosynthesis of proteins leading to, for example, increased mitochondrial volume (Pilegaard et al. 2000; Mahoney et al. 2005; Perry et al. 2010; Neufer et al. 2015). However, cellular homeostasis and preservation of muscle function also requires efficient degradation of dysfunctional cellular components through autophagy (Sandri 2010; Yan et al. 2012).

Activation of autophagy requires a sequence of multiple signaling events to form a phagophore membrane, which is maturated into an autophagosome allowing lysosomal degradation of proteins and other cellular components. Phagophore formation involves activation of the UNC‐51 like autophagy activating kinase (ULK)1, which forms a complex with several other autophagy‐related proteins resulting in phosphorylation of Beclin‐1 and subsequent maturation of the autophagosome (Hosokawa et al. 2009). Elongation of the autophagosome and targeting of proteins and organelles for degradation require the lipidation of microtubule‐associated protein 1 light chain 3 (LC3)I to LC3II (Kabeya et al. 2004).

Studies have reported that exercise activates autophagy, as shown by the conversion of LC3I to LC3II and reduced content of the autophagy adaptor protein p62 (Grumati et al. 2011; He et al. 2012). In accordance, it has been reported that exercise training increases basal autophagy flux and that autophagy is required for complete exercise training‐induced adaptations in angiogenesis, mitochondrial content, and endurance running capacity (Lira et al. 2013). Furthermore, oxidative muscle types have been shown to exhibit higher protein content of autophagy markers and higher autophagy flux than glycolytic muscle (Lira et al. 2013), suggesting that the muscle oxidative phenotype may be linked with autophagy capacity. The energy sensor 5′ AMP‐activated protein kinase (AMPK) is activated in skeletal muscle by a single exercise bout (Winder and Hardie 1996) and has been suggested to be involved in activation of autophagy through ULK1 Ser317 phosphorylation, while ULK1 Ser757 phosphorylation by mammalian target of rapamycin complex (mTORC)1 has been suggested to inhibit autophagosome formation (Kim et al. 2011). However, the regulatory mechanisms behind exercise‐induced activation of autophagy remain unclear.

Previous studies have identified the transcriptional co‐activator peroxisome proliferator‐activated receptor‐*γ* co‐activator (PGC)‐1*α* as a potent regulator of both basal and exercise training‐induced mitochondrial biogenesis (Wu et al. 1999; Baar et al. 2002; Pilegaard et al. 2003; Geng et al. 2010; Ringholm et al. 2013). Hence, PGC‐1*α* has been shown to be important for exercise‐induced mRNA responses (Leick et al. 2008) and exercise training‐induced adaptive increases in the content of metabolically related proteins (Geng et al. 2010; Ringholm et al. 2013), although PGC‐1*α* may not always be mandatory for exercise training‐induced increases in mitochondrial protein content (Leick et al. 2008). Recent studies have indicated that PGC‐1*α* may also play a role in basal (Lira et al. 2013) and exhaustive exercise‐induced (Vainshtein et al. 2015) autophagy. However, no studies to date have examined the time course of the regulation of autophagy during recovery from a single intensity/duration matched exercise bout and the role of muscle‐specific manipulation of the PGC‐1*α* gene in this. Therefore, the aim of this study was to examine the acute exercise‐induced autophagy response in skeletal muscle at several time points during recovery in mouse models with transgenic muscle‐specific overexpression (TG) and muscle‐specific knockout (MKO) of PGC‐1*α*.

## Methods

### Ethics statement

Animal experiments were approved by the Danish National Animal Experiment Inspectorate (2008/561‐1464) and performed in compliance with the European convention for the protection of vertebrate animals used for experiments and other scientific purposes (Council of Europe, no. 123, Strasbourg, France, 1985).

### Mice

Generation and characterization of the PGC‐1*α* MKO and TG mouse strains used in this study have been described elsewhere (Lin et al. 2002; Geng et al. 2010; Olesen et al. 2012). Littermate MKO and lox/lox control mice were bred by crossing heterozygous Myo‐Cre, lox/lox mice with lox/lox mice. Littermate TG and WT mice were bred by crossing heterozygous TG with WT mice. Genotypes of the mice were determined by PCR‐based genotyping, as previously described (Leick et al. 2008). Furthermore, efficient knockout of PGC‐1*α* in skeletal muscle in the MKO strain was verified by determining PGC‐1*α* mRNA content in quadriceps using real‐time PCR. Mice were kept on a 12:12 h light/dark cycle and had free access to water and standard rodent chow (Altromin no. 1324; Brogården, Lynge, Denmark).

### Experimental protocol

Twelve weeks old male MKO and TG mice and their respective littermate controls were subjected to a single 1 h treadmill running bout at 14 m/min and 10° incline (Exer‐4; Columbus Instruments, OH). Mice were euthanized by cervical dislocation immediately after and at 2, 6, and 10 h after running, while control mice rested for 1 h on the treadmill (*n* = 8–10) and were killed at the same time points as the exercise mice. Trunk blood was collected following decapitation and quadriceps muscles were quickly excised and frozen in liquid nitrogen. Plasma was obtained from blood samples by centrifugation at 2600 ***g*** for 15 min at 4°C. Samples were stored at −80°C until analyses.

### Plasma analyses

Plasma glucose concentration was determined on a fluorometer (Fluoroskan Ascent; Thermo Fisher Scientific, Waltham, MA) using NADH autoflourescence as previously described (Lowry and Passonneau 1972).

### Muscle glycogen

Glycogen content was determined in ~15 mg of quadriceps tissue as glycosyl units after acid hydrolysis using a fluorometer (Fluoroskan Ascent; Thermo Fisher Scientific) as previously described (Lowry and Passonneau 1972).

### RNA isolation, reverse transcription, and real‐time PCR

Quadriceps tissue was crushed in liquid nitrogen and ~20–25 mg was weighed out for RNA isolation. Total RNA was extracted using an adapted guanidinium thiocyanate–phenol–chloroform method as previously described (Pilegaard et al. 2000). Pelleted RNA extracts were resuspended in 0.1 mmol/L EDTA in DEPC‐treated H_2_O and RNA purity and concentration was determined by spectrophotometry (Nanodrop 1000; Thermo Fisher Scientific). Reverse transcription of mRNA to cDNA was performed using Superscript II and Oligo dT (Invitrogen, Carlsbad, CA) as previously described (Pilegaard et al. 2000). Real‐time PCR was performed using a QuantStudio 7 Flex Real‐Time PCR System (Applied Biosystems, Waltham, MA). Primers and 5′‐FAM and 3′‐TAMRA‐labeled TaqMan probes were designed using Primer Express 3.0 software (Applied Biosystems). Primers and TaqMan probes were obtained from TAG Copenhagen (Copenhagen, Denmark). Primer and probe sequences used were: PGC‐1*α* FP: 5′ CTC CCT TGT ATG TGA GAT CAC GTT 3′, PGC‐1*α* RP: 5′ TGC GGT ATT CAT CCC TCT TGA 3′, PGC‐1*α* probe: 5′ ACA GCC GTA GGC CCA GGT ACG ACA 3′, LC3 FP: 5′ CGA GCT CAT CAA GAT AAT CAG AC 3′, LC3 RP: 5′ TTC CTC CTG GTG AAT GGG C 3′, LC3 probe: 5′ CGC TTG CAG CTC AAT GCT AAC CAA GC 3′. Real‐time PCR was performed in triplicates with a total reaction volume of 10 *μ*L using Universal Mastermix II (Applied Biosystems). The cycle threshold was converted to a relative amount of target mRNA from a standard curve generated from a dilution series of a sample pool from all cDNA samples within each strain. Relative target mRNA content was normalized to total ssDNA content determined in each sample using OliGreen reagent (Molecular Probes, Leiden, The Netherlands) as previously described (Lundby et al. 2005).

### Muscle lysate and protein determination

Muscle lysate was generated from ~20 to 25 mg wet weight of crushed quadriceps tissue as previously described (Pilegaard et al. 2006). Supernatant was collected following centrifugation for 20 min at 16,000 ***g*** (4°C) and total protein content in the lysates was measured using the bicinchoninic acid assay (Thermo Fisher Scientific).

### SDS‐PAGE and western blotting

Relative content of target proteins was measured in muscle lysates by SDS‐PAGE and western blotting. Equal amounts of total protein were loaded from each sample, and samples from each group and genotype were distributed on each gel. Primary antibodies were used to detect ACC2 (streptavidin–HRP; Dako, Glostrup, Denmark), phospho‐ACC Ser212 (#07‐303; Millipore, Bedford, MA), AMPK*α*2 (provided by Graham Hardie, Dundee University, Dundee, UK), phospho‐AMPK Thr172 (#2535), p38 (#9212), phospho‐p38 Thr180/Tyr182 (#4631), LC3A/B (#4108), p62 (#5114), phospho‐ULK1 Ser317 (#12753), phospho‐ULK1 Ser757 (#6888) (Cell Signaling, Danvers, MA). Membrane imaging and band quantification were performed using ImageQuant LAS 4000 (GE Healthcare, Little Chalfont, UK) and ImageQuant TL v8.1 software (GE Healthcare). Protein content is expressed in arbitrary units and relative to a sample pool loaded on each side of all gels.

### Statistical analyses

Two‐way ANOVA was used to test the effect of a single exercise bout and genotype on mRNA, proteins, phosphorylations, glycogen content, protein carbonyl content and plasma glucose. In addition, one‐way ANOVA was used to determine the effect of exercise within each genotype separately. When main effects were observed, pairwise differences were determined using Student Newman–Keuls multiple comparisons test. When equal variance test failed, values were log transformed before analysis. *P* < 0.05 was considered significant and 0.05 ≤ *P* < 0.1 was interpreted as a tendency for a significant difference. Statistical analyses were performed using Sigmaplot 12.5 software (SYSTAT, Chicago, IL). All values are presented as mean ± SE.

## Results

### Plasma glucose and muscle glycogen

Plasma glucose concentration was unchanged throughout recovery relative to Rest in both genotypes in both mouse strains. There were no genotype difference in the MKO strain; however, TG mice had an overall higher plasma glucose concentration than WT (Table 1).

**Table 1:** PGC‐1*α* mRNA content, quadriceps glycogen content and plasma glucose concentration in PGC‐1*α* MKO and PGC‐1*α* TG mice and their respective controls (lox/lox and WT) at different time points after a 1 h bout of treadmill running

	Rest	0 h	2 h	6 h	10 h
PGC‐1*α* mRNA/ssDNA (AU)					
Lox/lox	0.2 ± 0.1	0.2 ± 0.0	0.4 ± 0.1	1.0 ± 0.2^a^	0.5 ± 0.1
Muscle glycogen (mmol/kg)					
Lox/lox	17.9 ± 1.8	13.0 ± 1.4^a^	20.1 ± 1.4	20.8 ± 1.1	19.5 ± 1.9
MKO	15.4 ± 1.3^b^	10.0 ± 1.0^b^, ^a^	16.3 ± 1.8^b^	18.5 ± 1.2^b^	16.9 ± 1.5^b^
Plasma glucose (mmol/L)					
Lox/lox	8.4 ± 0.6	7.7 ± 0.5	8.6 ± 0.3	7.5 ± 0.3	6.4 ± 0.2
MKO	6.9 ± 0.8	8.1 ± 0.4	8.1 ± 0.7	7.8 ± 0.5	7.3 ± 0.4
PGC‐1*α* mRNA/ssDNA (AU)					
WT	0.1 ± 0.0	0.1 ± 0.0	0,5 ± 0,1^a^	0.2 ± 0.1	0.2 ± 0.0
TG	1.6 ± 0.1^b^	2.0 ± 0.3^b^	1.6 ± 0.3^b^	1.4 ± 0.2^b^	1.5 ± 0.2^b^
Muscle glycogen (mmol/kg)					
WT	19.4 ± 1.3	11.2 ± 0.9^a^	20.3 ± 1.9	25.3 ± 1.3	25.6 ± 2.0
TG	35.7 ± 2.0^b^	33.4 ± 2.1^b^	36.3 ± 2.5^b^	45.7 ± 0.9^b^	43.0 ± 2.0^b^
Plasma glucose (mmol/L)					
WT	7.4 ± 0.3	7.8 ± 0.7	8.2 ± 0.6	7.8 ± 0.5	6.9 ± 0.4
TG^b^	8.4 ± 0.5	9.6 ± 0.5	7.5 ± 0.4	8.6 ± 0.1	8.4 ± 0.5

Values are mean ± SE with *n* = 6–9.

a Significantly different from Rest within genotype (*P* < 0.05).

b Significantly different from lox/lox or WT (*P* < 0.05).

Muscle glycogen content was 30–40% lower (*P* < 0.05) in WT/lox/lox mice immediately after the exercise bout than at rest. In addition, muscle glycogen content was 40–60% higher (*P* < 0.05) in TG than WT at all time points, while muscle glycogen was ~10% lower (*P* < 0.05) in MKO than in lox/lox mice (Table 1).

### PGC‐1*α* mRNA

To verify the efficiency of the single exercise bout in inducing an adaptive response, muscle PGC‐1*α* mRNA content was measured. In WT/lox/lox mice, the muscle PGC‐1*α* mRNA content was transiently elevated ~fourfold (*P* < 0.05) at 2 and 6 h after exercise relative to Rest. In TG mice, the PGC‐1*α* mRNA content was 3–7 fold higher (*P* < 0.05) than in WT at all time points (Table 1).

### Autophagy proteins

**Figure 1:**
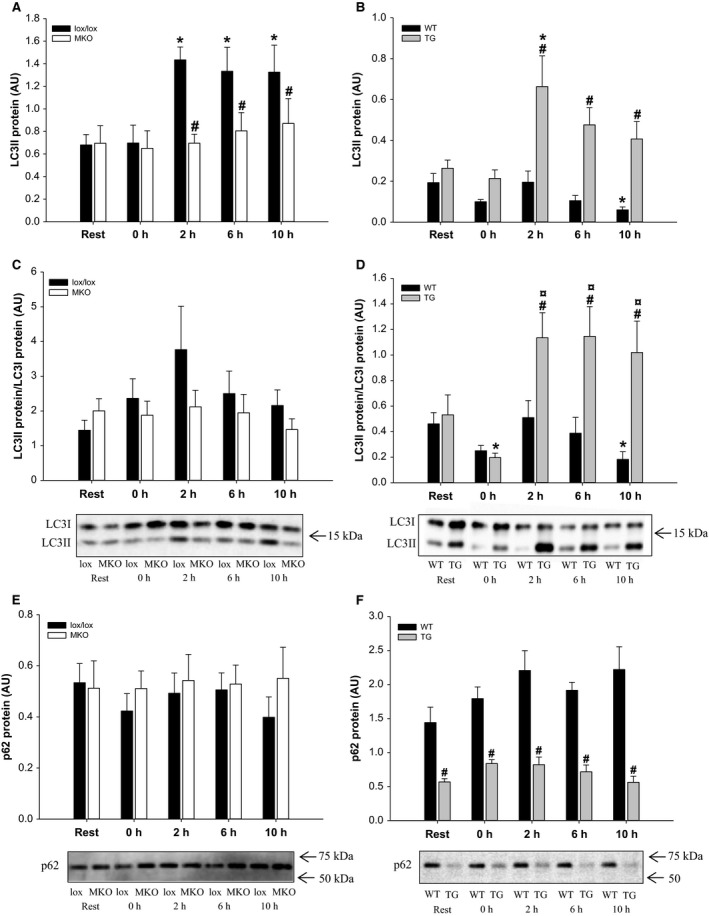
Quadriceps LC3II, LC3II/I, and p62 protein content and representative western blots from muscle‐specific PGC‐1*α* KO (MKO) (A, C and E) and muscle‐specific transgenic PGC‐1*α* overexpression (TG) mice (B, D, and F) and their respective littermate controls (lox/lox and WT), at Rest and 0, 2, 6, and 10 h after a single 1 h bout of treadmill running. Values are mean ± SE (*n* = 8–10). *Significantly different from Rest within given genotype (*P* < 0.05). ¤Significantly different from 0 h within given genotype (*P* < 0.05). #Significantly different from lox/lox or WT within given time point (*P* < 0.05).

As markers of autophagy, LC3 and p62 protein content was determined. In the PGC‐1*α* MKO strain, skeletal muscle LC3II protein content was ~twofold higher (*P* < 0.05) at 2, 6, and 10 h of recovery than at Rest in lox/lox, but not in MKO mice (Fig. 1A). Furthermore, LC3I+II protein content tended to be higher (*P* = 0.073) at 2 h after exercise than immediately after in lox/lox, but not MKO mice (data not shown). LC3II and total LC3 protein content was approximately 50% lower (*P* < 0.05) in MKO than lox/lox mice at 2 h of recovery. No differences were observed in the LC3II/I ratio or p62 protein content with exercise or genotype (Fig. 1C and E).

The content of LC3II and the LC3II/I ratio were lower (*P* < 0.05) at 10 h of recovery than at Rest in WT mice in the PGC‐1*α* TG strain. Furthermore, a single exercise bout resulted in a threefold higher (*P* < 0.05) LC3II protein content at 2 h of recovery than at Rest and a twofold higher (*P* < 0.05) LC3II/LC3I ratio at 2–10 h than immediately after exercise in TG, but not WT mice. LC3II and the LC3II/LC3I ratio were 2.5–5 fold higher (*P* < 0.05) in TG than WT at 2–10 h of recovery (Fig. 1B and D). LC3I+II protein content was unaffected by exercise in both PGC‐1*α* TG and WT, but ~twofold higher (*P* < 0.05) in PGC‐1*α* TG than WT immediately and 2 h after the exercise bout (data not shown). In addition, protein content of p62 was unaffected by exercise, but 50–70% lower (*P* < 0.05) in muscle from PGC‐1*α* TG than WT mice at all time points (Fig. 1F). No time or genotype differences were observed in LC3 mRNA content in either mouse strain (data not shown).

### AMPK and ACC

**Figure 2:**
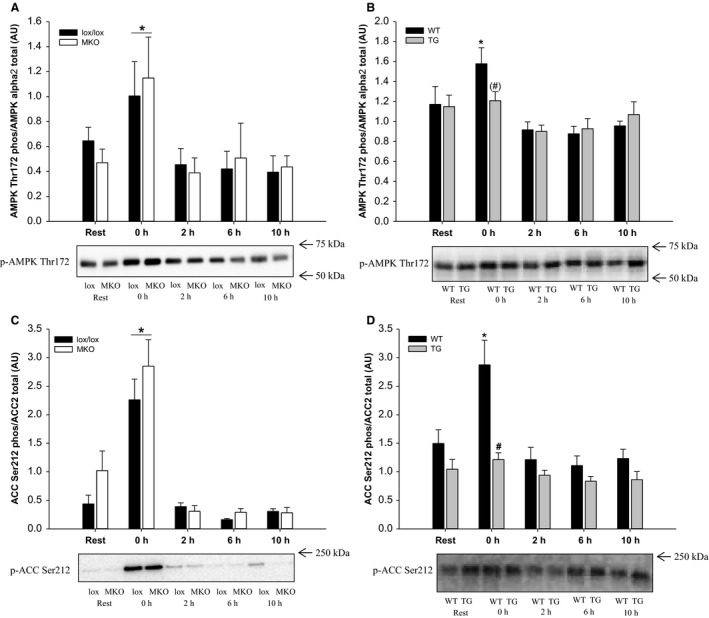
Quadriceps AMPK Thr172 phosphorylation and ACC2 Ser212 phosphorylation normalized to AMPK *α*2 and ACC2 protein content and representative western blots from muscle‐specific PGC‐1*α* KO (MKO) (A and C) and muscle‐specific transgenic PGC‐1*α* overexpression (TG) mice (B and D) and their respective littermate controls (lox/lox and WT), at Rest and 0, 2, 6, and 10 h after a single 1 h bout of treadmill running. Values are mean ± SE (*n* = 8–10). *Significantly different from Rest within given genotype (*P* < 0.05). ^#^Significantly different from lox/lox or WT within given time point (*P* < 0.05). Parentheses indicate a tendency for a significant difference (0.05 ≤ *P* < 0.10).

AMPK signaling was evaluated by measuring phosphorylation of AMPK at Thr172 and by Ser212 phosphorylation of ACC2. Immediately after the exercise bout, AMPK phosphorylation was ~1.5–2 fold higher (*P* < 0.05) in both lox/lox and MKO mice than at Rest, while ACC2 phosphorylation was ~three to four fold higher (*P* < 0.05) in both lox/lox and MKO mice than at Rest. No genotype differences were observed in AMPK and ACC2 phosphorylation in the PGC‐1*α* MKO strain (Fig. 2A and C).

In the PGC‐1*α* TG strain, AMPK and ACC2 phosphorylation was increased ~30% and twofold (*P* < 0.05), respectively, immediately after exercise in WT, but not TG mice (Fig. 2B and D). Phosphorylation of p38 (Thr180/Tyr182) was higher immediately after the exercise bout than at rest in all mice (data not shown).

### ULK1 phosphorylation

**Figure 3:**
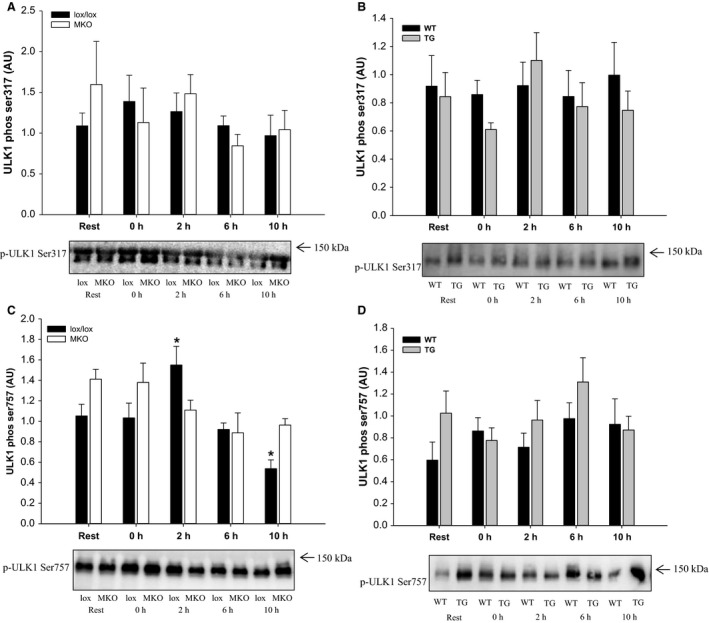
Quadriceps ULK1 phosphorylation (Ser317 and Ser757) and representative western blots from muscle‐specific PGC‐1*α* KO (MKO) (A and C) and muscle‐specific transgenic PGC‐1*α* overexpression (TG) mice (B and D) and their respective littermate controls (lox/lox and WT), at Rest and 0, 2, 6, and 10 h after a single 1 h bout of treadmill running. Values are mean ± SE (*n* = 8–10). *Significantly different from Rest within given genotype (*P* < 0.05). ^#^Significantly different from lox/lox or WT within given time point (*P* < 0.05). Parentheses indicate a tendency for a significant difference (0.05 ≤ *P* < 0.10).

To evaluate the autophagy‐related signaling, the phosphorylation level of ULK1 (Ser317 and Ser757) was measured. In the PGC‐1*α* MKO strain, ULK1 Ser317 phosphorylation was unaffected by exercise and genotype, while ULK1 Ser757 phosphorylation was transiently increased (*P* < 0.05) at 2 h into recovery in lox/lox, but not in MKO mice (Fig. 3A and C).

In the PGC‐1*α* TG strain, phosphorylation of ULK1 was unaffected by exercise in both WT and TG mice with no differences observed between genotypes at any time point (Fig. 3B and D).

### Protein carbonyl content

**Figure 4:**
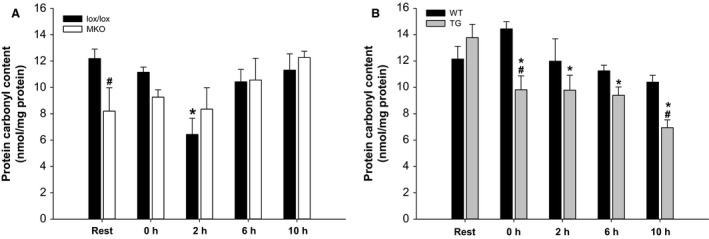
Quadriceps protein carbonyl content from muscle‐specific PGC‐1*α* KO (MKO) (A) and muscle‐specific transgenic PGC‐1*α* overexpression (TG) mice (B) and their respective littermate controls (lox/lox and WT), at Rest and 0, 2, 6, and 10 h after a single 1 h bout of treadmill running. Values are mean ± SE (*n* = 8–10). *Significantly different from Rest within given genotype (*P* < 0.05). ^#^Significantly different from lox/lox or WT within given time point (*P* < 0.05).

Protein carbonyl content was determined as a marker of total protein oxidation. Basal protein carbonyl content was ~30% lower (*P* < 0.05) in MKO than in lox/lox mice. Protein carbonyl content was transiently lower (*P* < 0.05) at 2 h into recovery than at Rest in lox/lox, but not in MKO mice (Fig. 4A).

In TG mice, protein carbonyl content was ~30–50% lower (*P* < 0.05) at all time points during recovery from exercise than at Rest, whereas exercise did not affect protein carbonyl content in WT mice. Protein carbonyl content was overall lower (*P* < 0.05) in TG than in WT mice; an effect that was localized to 0 and 10 h into recovery (Fig. 4B).

## Discussion

The main findings of this study are that PGC‐1*α* is required for exercise‐induced autophagy in mouse skeletal muscle, as shown by increased lipidation of LC3 from 2 h of recovery after a single prolonged exercise bout in lox/lox, but not in MKO mice, while a similar response was observed in TG mice. The elevated LC3II protein content during recovery from exercise was not associated with changes in p62 protein content or ULK1 Ser317 phosphorylation. However, lower protein carbonyl content was observed in lox/lox and TG mice after exercise coinciding with the increased LC3 lipidation, suggesting that activation of autophagy in response to exercise may serve to remove oxidatively damaged proteins.

This study is the first to examine the role of muscle‐specific manipulation of the PGC‐1*α* gene in acute exercise‐induced responses of proteins involved in autophagy at several time points during the recovery period. The observed transient increase in PGC‐1*α* mRNA content in lox/lox and WT mice after the 1 h treadmill running bout confirms that the single exercise bout was sufficient to induce an adaptive gene response during the recovery period similar to previous studies (Pilegaard et al. 2003; Perry et al. 2010). This was further supported by the reduced muscle glycogen concentration and elevated AMPK, ACC, and p38 phosphorylation observed immediately after the exercise bout. The conversion of LC3I to LC3II is the most widely used marker for activation of autophagy (Mizushima and Yoshimori 2007; Grumati et al. 2011; He et al. 2012; Jamart et al. 2013), although the most appropriate way to interpret LC3 immunoblots is unclear. Because the sensitivity of LC3 antibodies may be higher for LC3II than LC3I, comparing ratios between LC3II and LC3I may exaggerate potential effects, and therefore it has been argued that the most appropriate way to assess autophagy is by comparing LC3II amount between samples (Mizushima and Yoshimori 2007). The present finding that acute exercise increased the content of LC3II in skeletal muscle from lox/lox mice is similar to several previous observations (Grumati et al. 2011; He et al. 2012; Jamart et al. 2013; Vainshtein et al. 2015). In addition, the findings that LC3II content was higher in lox/lox compared with MKO mice and in TG compared with WT mice from 2 h into recovery from exercise, suggest that PGC‐1*α* promotes the exercise‐induced autophagy response. However, whether this is due to direct transcriptional regulation of factors involved in the autophagy response, or due to the altered metabolic phenotype in MKO and TG mice is unclear. It has been reported that basal levels of key autophagy proteins is higher in oxidative versus glycolytic muscle in mice (Lira et al. 2013), suggesting that mitochondrial bioenergetics may be related to the augmented exercise‐induced autophagy response observed in lox/lox versus MKO and TG versus WT mice, respectively. The finding that LC3II protein content did not increase in response to exercise in WT mice in the PGC‐1*α* TG strain suggests that a given acute exercise protocol may not elicit induction of autophagy in all mouse strains. This is supported by the report that large genetic and physiological variability exists between different inbred mouse strains (Lerman et al. 2002), which may affect the autophagic response to exercise. Indeed, it has been shown that exercise‐induced autophagy in mouse skeletal muscle is highly dependent on for example nutritional state (Jamart et al. 2013), supporting that metabolic differences between mouse strains may be of importance for the exercise‐induced autophagy response. In this study, all mice were exercised at the same absolute intensity, and it can therefore not be excluded that the differential responses observed between the MKO and TG strains are due to exercise intensity‐specific regulation of the autophagy response. In human skeletal muscle, exercise‐induced autophagy has recently been reported to be dependent on exercise intensity (Schwalm et al. 2015). However, because TG mice have a higher endurance exercise capacity than WT (Calvo et al. 2008), the present observation that TG mice exhibited a larger exercise‐induced autophagy response than WT at the same absolute intensity, suggests that exercise‐induced autophagy in skeletal muscle may not always be intensity dependent, at least not with forced overexpression of PGC‐1*α*. Therefore, the role of PGC‐1*α* in the intensity dependency of exercise‐induced autophagy activation in skeletal muscle should be investigated further.

The present finding that p62 content was unchanged by exercise in both mouse strains is consistent with recent observations (Jamart et al. 2013; Vainshtein et al. 2015). However, others have reported lower p62 content in response to exercise (He et al. 2012) in an intensity‐dependent manner (Schwalm et al. 2015), which was interpreted as a result of lysosomal degradation of p62. These inconsistent findings may be due to relatively small amounts of total p62 content being degraded during autophagy or due to a high turnover rate of p62. This is supported by a recent mouse study showing that prior treatment with the lysosomal inhibitor colchicine resulted in p62 accumulation in the skeletal muscle mitochondrial fraction after exercise, while p62 protein content remained constant without autophagy blockade (Vainshtein et al. 2015). The finding that p62 was similar in lox/lox and MKO muscle at all time points is in accordance with observations from whole body PGC‐1*α* KO mice (Vainshtein et al. 2015), and the lower p62 content in PGC‐1*α* TG mice than in WT observed in this study is also consistent with previous observations in the same mouse strain (Lira et al. 2013). The reduced skeletal muscle p62 content observed in TG mice has been interpreted as a result of increased autophagic flux, when PGC‐1*α* content is elevated (Lira et al. 2013), which is consistent with the present finding that exercise‐induced autophagy was enhanced by overexpression of PGC‐1*α*. Thus, PGC‐1*α* seems to promote exercise‐induced autophagy in skeletal muscle, but may not be required for basal autophagy flux.

A role of AMPK in regulating autophagy through phosphorylation and activation of the autophagy‐initiating kinase ULK1at Ser317 has been proposed, while ULK1 Ser757 phosphorylation by mTORC1 has been reported to inhibit autophagosome formation in vitro (Kim et al. 2011). Recent studies have reported a relationship between exercise‐induced AMPK activation and ULK1 phosphorylation in humans (Moller et al. 2015; Schwalm et al. 2015); however, it has not been determined whether AMPK is required for exercise‐induced autophagy. In this study, exercise‐induced phosphorylation of AMPK and its downstream target ACC was similar in lox/lox and MKO mice, while TG muscle did not exhibit increased AMPK or ACC phosphorylation in response to the exercise bout. The finding that ULK1 Ser317 phosphorylation was unaffected by exercise in both mouse strains, is in accordance with a previous report (Jamart et al. 2013), suggesting that AMPK‐dependent phosphorylation of ULK1 at Ser317 is not required for exercise‐induced autophagy. Supporting the dissociation between exercise‐induced autophagy and AMPK activation is the previous observation that exercise‐induced autophagy was attenuated in whole‐body PGC‐1*α* KO mice although AMPK activity was completely preserved (Vainshtein et al. 2015).

Reactive oxygen and nitrogen species (ROS/RNS) are known to be produced during exercise with the potential to cause irreversible damage to organelles and proteins (Powers et al. 2011). Many processes leading to increased ROS production are also known to induce autophagy, suggesting a link between redox signaling and autophagy (Lee et al. 2012). In accordance, a recent study showed higher basal content of protein carbonyls in autophagy deficient mouse muscle (Lo et al. 2014), suggesting that autophagy is important for degradation of oxidized proteins. The present finding that protein carbonyl content was lower after exercise only in lox/lox and TG mice, coinciding with the increased LC3II amount, suggests that activation of autophagy may be a mechanism for targeted removal of oxidatively damaged proteins during recovery from exercise. Future studies on the role of autophagy in exercise‐induced redox signaling and maintenance of redox balance are therefore warranted. Interestingly, a lower protein carbonyl content was observed in skeletal muscle from MKO than lox/lox mice, which is in contrast with the increased protein carbonyl content found in skeletal muscle of whole‐body PGC‐1*α* KO mice (Olesen et al. 2013), suggesting that there may be PGC‐1*α*‐dependent changes in nonmuscle tissue, which could affect the formation or degradation of protein carbonyls in skeletal muscle.

In conclusion, this study provides evidence that a component of autophagy activation in skeletal muscle during recovery from a single exercise bout is dependent on the level of PGC‐1*α* present in the muscle. The observed exercise‐induced autophagy response did not seem to be associated with AMPK‐mediated phosphorylation of ULK1. However, the PGC‐1*α*‐dependent reduction of protein carbonyl content after exercise revealed an association between exercise‐induced autophagy and reduced protein oxidation. Thus, the present results suggest a role of PGC‐1*α* in coordinating exercise‐induced mitochondrial biogenesis and autophagic removal of damaged cellular components.

## Conflict of Interest

The authors declare no conflict of interests.
